# Supplementary material for: Reconciling Mining with the Conservation of Cave Biodiversity: A Quantitative Baseline to Help Establish Conservation Priorities
Source: PLoS One. 2016 Dec 20;11(12):e0168348. doi: 10.1371/journal.pone.0168348 (PMC5173368; doi:10.1371/journal.pone.0168348)
Supplement: S1 Dataset — (ZIP) [file pone.0168348.s002.zip › Taxa/Serra Norte/SN_2013/N4WS/Lista N4WS-10.pdf]

CAVIDADE N4WS-0010

Classe	Ordem	Fam/Outros	Gên/Outros	Espécie	S-U	U-S	Zon.
Annelida	Oligochaeta			sp.		1	P
Arachnida	Acari	(Parasitengonina)		sp.2	X		P
Arachnida	Acari	(Parasitengonina)		sp.3		X	P
Arachnida	Acari	(Parasitengonina)		sp.5	X	X	P
Arachnida	Acari	Acaridae		sp.2	X	X	P
Arachnida	Acari	Argasidae	*Ornithodoros*	sp.1	X	X	E/P
Arachnida	Acari	Cheyletidae		sp.1		X	E
Arachnida	Acari	Rhagidiidae		sp.1		X	P
Arachnida	Amblypygi	Phrynidae	*Heterophrynus*	*longicornis*	1		E
Arachnida	Araneae	Araneidae	*Alpaida*	*septemmammata*	X		P
Arachnida	Araneae	Ctenidae		jovem	1	1	E/P
Arachnida	Araneae	Dipluridae		jovem		1	E
Arachnida	Araneae	Gnaphosidae		jovem	X		P
Arachnida	Araneae	Ochyroceratidae	*Speocera*	sp.1	X	X	P
Arachnida	Araneae	Oonopidae	aff. *Matiotia*	sp.1	X		P
Arachnida	Araneae	Pholcidae	*Mesabolivar*	*aurantiacus*	X	X	E/P
Arachnida	Araneae	Salticidae	*Amphidraus*	sp.1	X	X	E
Arachnida	Araneae	Scytodidae	*Scytodes*	*eleonorae*		4	P
Arachnida	Araneae	Scytodidae	*Scytodes*	*globula*	16	10	P
Arachnida	Araneae	Theraphosidae		jovem	7	1	P
Arachnida	Araneae	Theridiidae		jovem		X	E/P
Arachnida	Araneae	Theridiosomatidae	*Plato*	sp.1	X	X	E/P
Arachnida	Araneae	Theridiosomatidae	*Plato*	sp.3	X		E
Arachnida	Opiliones	Escadabiidae		sp.2		X	P
Arachnida	Opiliones	Sclerosomatidae		jovem		X	E
Arachnida	Pseudoscorpiones	Chernetidae	*Spelaeochernes*	sp.1	X	X	E/P
Chilopoda	Geophilomorpha	Geophilidae	*Hyphydrophilus*	sp.1	1		P
Chilopoda	Geophilomorpha	Geophilidae		sp.2	1		P
Chilopoda	Scolopendromorpha	Scolopendridae	*Rhoda*	sp.1		1	P
Diplopoda	Polydesmida	Chelodesmidae		sp.2		1	P
Diplopoda	Polydesmida	Pyrgodesmidae		sp.1		X	P
Entognatha	Collembola	Cyphoderidae		sp.1		X	P
Insecta	Blattodea	Blaberidae		jovem	4		P
Insecta	Blattodea	Blattellidae		jovem	1		P
Insecta	Blattodea	Polyphagidae		jovem	2		P
Insecta	Coleoptera	Curculionidae		jovem	X		P
Insecta	Coleoptera	Tenebrionidae		jovem	X		P
Insecta	Diptera	Cecidomyiidae	Cecidomyiinae	sp.		X	P
Insecta	Diptera	Ceratopogonidae		sp.	X		P
Insecta	Diptera	Culicidae	*Culex*	sp.		X	P
Insecta	Diptera	Drosophilidae	*Drosophila*	*eleonorae*		X	P
Insecta	Diptera	Ephydridae		sp.		X	E
Insecta	Diptera	Psychodidae	*Pintomyia*	*gruta*	X		P
Insecta	Diptera	Psychodidae	*Sciopemyia*	*sordellii*		X	E/P
Insecta	Diptera	Tipulidae	Tipulinae	sp.	X		P
Insecta	Heteroptera	Reduviidae	Reduviinae	jovem	22	9	E/P
Insecta	Hymenoptera	Bethylidae		sp.1	X		P
Insecta	Hymenoptera	Formicidae	*Solenopsis*	sp.2		X	E/P
Insecta	Hymenoptera	Pemphredonidae		sp.1		X	E

Insecta	Isoptera	Termitidae	*Nasutitermes*	sp.		X	E/P
Insecta	Lepidoptera	Noctuoidea		sp.1	1	6	E
Insecta	Lepidoptera	Noctuoidea		sp.2		X	E
Insecta	Lepidoptera	Tineoidea		sp.6	X	X	E/P
Insecta	Neuroptera	Myrmeleontidae		jovem	7	1	E/P
Insecta	Orthoptera	Mogoplistidae		sp.		3	P
Insecta	Orthoptera	Phalangopsidae	*Phalangopsis*	sp.	37	89	P
Insecta	Orthoptera	Phalangopsidae		sp.4		4	P
Insecta	Psocoptera	Psocomorpha		jovem		X	P
Insecta	Psocoptera	Psyllipsocidae	*Psocathropos*	sp.1		X	E
Insecta	Psocoptera	Psyllipsocidae	*Psyllipsocus*	sp.1	X	X	E/P
Gastropoda	Pulmonata	Bulimulidae	*Naesiotus*	sp.	X	X	P
Amphibia	Anura	Strabomantidae	*Pristimantis*	cf. *fenestratus*	1		E
Mammalia	Chiroptera	Emballonuridae	*Peropteryx*	sp.	7	20	E
Mammalia	Chiroptera	Furipteridae	*Furipterus*	*horrens*		2	P
Mammalia	Chiroptera	Phyllostomidae	Glossophaginae	sp.	4		P
Mammalia	Chiroptera	Phyllostomidae	*Trachops*	*cirrhosus*	4		P
Mammalia	Rodentia	Cricetidae	*Rhipidomys*	sp.		1	P
Reptilia	Squamata	Gekkonidae	*Thecadactylus*	sp.		1	E
